# Supplementary material for: Correcting market failure for no-regret electric road investments under uncertainty
Source: Nat Commun. 2025 Aug 11;16:7398. doi: 10.1038/s41467-025-62679-w (PMC12340124; doi:10.1038/s41467-025-62679-w)
Supplement: Supplementary file 1 — Supplementary Information [file 41467_2025_62679_MOESM1_ESM.pdf]

Correcting Market Failure for No-Regret Electric Road Investments under Uncertainty

Supplementary Information

Jakob Rogstadius[1*], Hampus Alfredsson[1], Henrik Sällberg[2], Karl-Filip Faxen[1]

[1] RISE Research Institutes of Sweden AB, Lindholmspiren 7A, 417 56 Göteborg, Sweden

[2] Blekinge Institute of Technology, Valhallavägen 10, 371 79 Karlskrona, Sweden

[*] Corresponding author, jakob.rogstadius@ri.se

This supplementary information contains all parameter settings used for the simulation model in the study. Table cells with numbers in *italic* indicate that the value has been derived from other values.

Supplementary Items List

Supplementary Table 1: World Parameter Settings 3

Supplementary Table 2: Battery Parameter Settings (Gross) 4

Supplementary Table 3: Powertrain Parameter Settings........ 5

Supplementary Table 4: BET vs. ICET Cost Differences (2020 Euro, excluding traction battery) 5

Supplementary Table 5: BET vs. ICET Weight Differences (excluding traction battery)........ 5

Supplementary Table 6: ICET Powertrain Cost (vs. 2020) 5

Supplementary Table 7: Vehicle Parameter Settings 6

Supplementary Table 8: HGV60 Parameter Settings 8

Supplementary Table 9: HGV40 Parameter Settings 9

Supplementary Table 10: MGV24 Parameter Settings........ 10

Supplementary Table 11: MGV16 Parameter Settings........11

Supplementary Table 12: Charging Infrastructure Parameter Settings........ 12

Supplementary Table 13: Infrastructure Utilization Curve Parameter Settings........ 14

Supplementary Figure 1: BET Purchase Cost 15

Supplementary Figure 2: ERS Cost........ 16

Supplementary Figure 3: Itemized system costs 17

Supplementary Figure 4: Infrastructure impact on levelized BEV costs........ 17

Supplementary Figure 5: Rest stop and ERS placement 18

Supplementary Figure 6: Effects of taking shorter and more frequent rest stops........ 19

Supplementary Method 1: Calculation of the build-out order of segments in the ERS network 20

Supplementary Table 1: World Parameter Settings

Values used for all input parameters in MOSTACHI's "World" category. While electricity price areas are supported, all areas/regions were given the same pricing for this study.

Subgroup	variable	unit	2020	2025	2030	2035	2040	2045	2050	Annual change	Source
Economy	Public sector interest rate	percent	2%	2%	2%	2%	2%	2%	2%	0%	https://www.pwc.se/sv/pdf-reports/corporate-finance/riskpremiestudien-2021.pdf, https://www.riksgalden.se/sv/var-verksamhet/statslanerantan/
Economy	Private sector interest public charging and trucks	percent	3%	3%	3%	3%	3%	3%	3%	0%	6% above base interest, companies with credit rating AAA/AA (https://firmalan.com/sv/lana-pengar/jamfor-lan/)
Economy	Private sector interest depot charging	percent	7%	7%	7%	7%	7%	7%	7%	0%	10% above base interest. Depot chargers should offer poor security. (https://firmalan.com/sv/lana-pengar/jamfor-lan/)
Economy	Heavy traffic volume vs 2020	percent	2.50	2.67	2.84	3.03	3.24	3.45	3.68	2%	The Swedish Transport Administration's base forecast for road cargo 2017-2040, Fig 6.4 (https://The Swedish Transport Administration.diva-portal.org/smash/get/diva2:1749263/FULLTEXT01.pdf)
Economy	Logistic BEV penalty	percent	15%	13%	10%	8%	5%	3%	0%	-2.5%	Guess based on reasoning that BEVs will be less flexible/viable on fewer routes in the beginning. Single-route levelized transport cost for BETs must beat diesel by this margin.
Charging	Extra stop overhead	h	0.25	0.21	0.18	0.16	0.14	0.12	0.10	-3%	Guess. Getting to the charger, plugging in, paying, battery conditioning, will get smoother.
CO_2	SCC	euro per kg	0.08	0.165	0.25	0.3875	0.525	0.6625	0.8	0%	https://www.eib.org/attachments/lucalli/20240105_eib_sasb_report_2023_en.pdf
CO_2	Tax	ratio of SCC	0.7	0.8	0.8	0.9	0.9	1.0	1.0	5%	To match current CO_2 tax.
FossilDiesel	Price	euro per liter	0.6	0.6	0.7	0.7	0.8	0.8	0.9	1%	Approximate product cost in Sweden in april 2023, estimated from product costs of FAME and HVO and blend ratios. Highly unreliable forecast.
RenewableDiesel	Price	euro per liter	1.50	1.50	1.59	1.69	1.79	1.90	2.02	-1%	alliance.eu/efuels/costs-outlook), which is likely a biased source.
RenewableDiesel	Supply cap	liter per year	175 000 000	193 214 141	213 324 023	235 526 959	260 040 794	287 106 049	316 988 277	2%	"Blend guess" ratio of total fuel used in 2020 according to the simulation.
RenewableDiesel	Blend guess	ratio	0.05	0.06	0.08	0.29	0.45	0.57	0.67	15%	https://www.energimyndigheten.se/globalassets/nyheter/2020/er-2020_26-drivmedel-2019.pdf
FossilDiesel	Emissions	kg CO_2 per liter	3.23	3.23	3.23	3.23	3.23	3.23	3.23	0%	Auke Hoekstra (2020) "Electric trucks: economically and environmentally desirable but misunderstood"
RenewableDiesel	Emissions	kg CO_2 per liter	0.67	0.52	0.40	0.31	0.24	0.19	0.14	-5%	2020 value from https://www.miljofordon.se/bilar/miljoepaaverkan/ Most of biodiesel's emission is from use of fossil oil, which is expected to gradually be displaced by sustainable sources.
Diesel	Price	euro per liter	0.65	0.69	0.75	1.00	1.23	1.44	1.64	n/a	
Diesel	Emissions	kg CO_2 per liter	3.10	3.07	3.00	2.39	1.89	1.48	1.16	n/a	
Diesel	CO_2 tax	euro per liter	0.17	0.38	0.60	0.79	0.89	0.93	0.93	n/a	CO_2 tax on diesel in Sweden in 2022.
Diesel	Pollution tax	euro per liter	0.020	0.032	0.052	0.084	0.135	0.217	0.349	10%	Guess
Diesel	Road tax	euro per liter	0.40	0.40	0.40	0.40	0.40	0.40	0.40	0%	Swedish energy tax has ranged between 1.5 and 2.5 SEK/liter. German energy tax is approx €0.5/liter.
Electricity	Conversion ratio	kWh per liter	4.2	4.2	4.2	4.2	4.2	4.2	4.2	n/a	
Electricity	Road tax	euro per kWh	0.096	0.096	0.096	0.096	0.096	0.096	0.096	n/a	
All regions	Price min	euro per kWh	0.071	0.071	0.071	0.071	0.071	0.071	0.071	0	Nordpool spot prices (DE-LU), min average price per hour, 2021
All regions	Price max	euro per kWh	0.125	0.125	0.125	0.125	0.125	0.125	0.125	0	Nordpool spot prices (DE-LU), max average price per hour, 2021
All regions	CO_2 emissions	kg per kWh	0.265	0.175	0.115	0.076	0.050	0.033	0.022	-8%	Greenhouse gas emission intensity of electricity generation in Europe (https://www.eea.europa.eu/ims/greenhouse-gas-emission-intensity-of-1)
All regions	CO_2 tax	euro per kWh	0.015	0.022	0.023	0.025	0.024	0.021	0.017	n/a	

Supplementary Table 2: Battery Parameter Settings (Gross)

Values used for all input parameters in MOSTACHI's "Batteries (gross)" category. Dominant battery chemistries are assumed to be unknown and to change over the 2020-2050 period and parameter estimates have therefore been made based on joint forecasts of combinations of higher-level performance characteristics. Battery parameters and their future development were first estimated based on information from Battery University (https://batteryuniversity.com) and Wikipedia's article on Lithium-ion batteries (https://en.wikipedia.org/wiki/Lithium-ion_battery). As parameters are in practice a chemical trade-off, the joint set of parameters was adjusted based on review by an anonymous truck manufacturer engineer and several experts in a public LinkedIn discussion (https://www.linkedin.com/posts/jakobrogstadius_please-find-problems-in-my-math-these-are-activity-6777180676291653632-jaJB/). The joint impact of all these parameters is that levelized battery costs become insignificant during the later simulation periods. Many parameters must be altered substantially for simulation outcomes to be affected at all.

variable	unit	2020	2025	*2030*	*2035*	*2040*	*2045*	*2050*	Annual change	Source
SoC window	ratio	0.65	0.83	*0.91*	*0.96*	*0.98*	*0.99*	*0.99*	1%	Truck OEM engineer (anonymous)
Calendar lifetime	years	15	17	*18*	*20*	*21*	*23*	*24*	0.3	See comment.
Cycle lifetime	cycles	1500	2 007	*2 686*	*3 595*	*4 811*	*6 438*	*8 615*	6%	See comment.
Reference charging rate	c	1	1.22	*1.48*	*1.80*	*2.19*	*2.67*	*3.24*	4%	See comment.
Reference discharging rate	c	1.5	1.82	*2.22*	*2.70*	*3.29*	*4.00*	*4.87*	4%	See comment.
Max permitted charging rate	c	*1.5*	*1.82*	*2.22*	*2.70*	*3.29*	*4.00*	*4.87*	n/a	1.5 * reference rate
Max permitted discharging rate	c	*2.25*	*2.74*	*3.33*	*4.05*	*4.93*	*6.00*	*7.30*	n/a	1.5 * reference rate
Specific energy	kWh per kg	0.2	0.23	*0.27*	*0.31*	*0.36*	*0.42*	*0.49*	3%	See comment.
Energy density	kWh per liter	0.5	0.58	*0.67*	*0.78*	*0.90*	*1.05*	*1.21*	3%	See comment.
Pack cost	€ per kWh	270	206	*159*	*125*	*100*	*81*	*68*	-5%	Link, S., Stephan, A., Speth, D. et al. Rapidly declining costs of truck batteries and fuel cells enable large-scale road freight electrification. Nat Energy 9, 1032–1039 (2024). https://doi.org/10.1038/s41560-024-01531-9.
Production emissions	kg CO_2 per kWh	150	99	*65*	*43*	*28*	*19*	*12*	-8%	Statement by Northvolt that emissions will be 25% of a Chinese competitor, claimed at 160-180. Trucks in Sweden are mostly Volvo and Scania, which will use Northvolt. Remaining emissions are from raw materials production.
End of life definition	ratio of gross	0.8	80%	*80%*	*80%*	*80%*	*80%*	*80%*	0%	See comment.

Supplementary Table 3: Powertrain Parameter Settings

Values used for all input parameters in MOSTACHI's "Powertrains" category. All values from The Swedish Transport Administration's 2021 report "Behov av laddinfrastruktur för snabbladdning av tunga fordon längs större vägar", with adjustments by Nils-Gunnar Vågstedt, Scania (spring 2021).

variable	unit	2020	2025	2030	2035	2040	2045	2050	Annual change	Source
Cost of replaced ICE powertrain	SEK/truck	500	*526*	*552*	*580*	*610*	*641*	*674*	1%	See caption
Cost of replaced ICE powertrain	SEK/kW	650	*683*	*718*	*755*	*793*	*834*	*876*	1%	See caption
Cost of new EV powertrain	SEK/truck	40 000	*36 157*	*32 683*	*29 543*	*26 704*	*24 139*	*21 819*	-2%	See caption
Cost of new EV powertrain	SEK/kW	190	*172*	*155*	*140*	*127*	*115*	*104*	-2%	See caption
Vehicle OEM markup	%	1.48	*1.48*	*1.48*	*1.48*	*1.48*	*1.48*	*1.48*	0%	See caption
ICE weight to power ratio	kg/kW	8.03	*8.03*	*8.03*	*8.03*	*8.03*	*8.03*	*8.03*	0%	See caption
BEV weight to power ratio	kg/kW	1.07	*1.07*	*1.07*	*1.07*	*1.07*	*1.07*	*1.07*	0%	See caption

Supplementary Table 4: BET vs. ICET Cost Differences (2020 Euro, excluding traction battery)

Derived vehicle cost differences for battery-electric trucks (BET), compared with internal combustion engine trucks (ICET) in the same vehicle class and model year.

vehicle class	kW	2020	2025	2030	2035	2040	2045	2050
HGV60	750	*-43 405*	*-49 434*	*-55 403*	*-61 345*	*-67 291*	*-73 270*	*-79 309*
HGV40	550	*-30 334*	*-34 901*	*-39 411*	*-43 889*	*-48 358*	*-52 841*	*-57 359*
MGV24	300	*-13 995*	*-16 736*	*-19 422*	*-22 069*	*-24 692*	*-27 305*	*-29 921*
MGV16	160	*-4 845*	*-6 563*	*-8 228*	*-9 850*	*-11 439*	*-13 005*	*-14 556*

Supplementary Table 5: BET vs. ICET Weight Differences (excluding traction battery)

Derived vehicle weight differences for battery-electric trucks (BET), compared with internal combustion engine trucks (ICET) in the same vehicle class and model year.

vehicle class	kW	2020	2025	2030	2035	2040	2045	2050
HGV60	750	*-5 220*	*-5 220*	*-5 220*	*-5 220*	*-5 220*	*-5 220*	*-5 220*
HGV40	550	*-3 828*	*-3 828*	*-3 828*	*-3 828*	*-3 828*	*-3 828*	*-3 828*
MGV24	300	*-2 088*	*-2 088*	*-2 088*	*-2 088*	*-2 088*	*-2 088*	*-2 088*
MGV16	160	*-1 114*	*-1 114*	*-1 114*	*-1 114*	*-1 114*	*-1 114*	*-1 114*

Supplementary Table 6: ICET Powertrain Cost (vs. 2020)

Derived progression of powertrain costs for internal combustion engine trucks (ICET), relative to ICETs in the same weight class in model year 2020.

vehicle class	kW	2020	2025	2030	2035	2040	2045	2050
HGV60	750	*0*	*3 537*	*7 254*	*11 161*	*15 267*	*19 582*	*24 118*
HGV40	550	*0*	*2 595*	*5 322*	*8 188*	*11 200*	*14 366*	*17 693*
MGV24	300	*0*	*1 417*	*2 906*	*4 471*	*6 116*	*7 845*	*9 662*
MGV16	160	*0*	*757*	*1 553*	*2 390*	*3 269*	*4 193*	*5 165*

Supplementary Table 7: Vehicle Parameter Settings

Values used for all input parameters in MOSTACHI's "Vehicles" category. ASEK 7 refers to the Swedish Transport Administration's reference tables "Analysmetod och samhällsekonomiska kalkylvärden", chapter 14 "Operativa trafikeringskostnader för godstransporter". Assumed annual reductions in energy consumption differ for diesel and electric trucks. In a diesel truck, approximately 65% of all the heat energy in the fuel goes to heat losses in the powertrain, while the remaining ~35% of energy is used to overcome aerodynamic drag, rolling resistance and potential energy from moving the vehicle weight up and down. For simplicity, we refer to the first as waste and the second as work. Historically, improvements have been made to both waste and work, each contributing approximately 0.5% reduction in total fuel consumption for new diesel trucks. Since work constitutes only ~35% of total fuel consumption, 0.5% reduction in total fuel consumption is equivalent to ~1.4% reduction in energy consumption for work. As truck manufacturers now redirect their R&D focus to electric powertrains, no further reductions are expected in terms of diesel powertrain waste, but improvements to the vehicle body continue as before. Total assumed reduction in fuel consumption for diesel trucks is thus 0.5% per year. In electric trucks, less waste means that the total energy consumption is much lower – the electrical energy used by an electric truck is only around 40% of the heat energy content of the diesel consumed by a traditional truck. Less waste also changes the relative share of total energy that goes to work, from ~35% to ~85%. Even without any assumed improvements to the electrical powertrain efficiency, this means that continued improvements to the vehicle body alone will yield at least 1.2% annual reduction in total energy consumption for electric trucks. Our assumed value of 1% reduction per year is thus conservative. In addition to marginal reductions in powertrain losses, there is potential for reduced charging losses, improved regeneration, thermal insulation of the cabin, more energy efficient on-board electronics, algorithms and control systems. Assumptions and reasoning are based on personal communication with Nils-Gunnar Vågstedt, Scania.

group	variable	unit	HGV60	HGV40	MGV24	MGV16	Annual change	Source
Common	Annual distance	km	90 000	115 000	80 000	42 000	0%	Values for Swedish-registered trucks, from The Swedish Transport Administration, "Behov av laddinfrastruktur för snabbladdning av tunga fordon längs större vägar", fig. 13
Common	Utilization	calendar days per year	230	230	230	230	0%	Informed guess
Common	Max time in transit	h per day	9	11	9	9	0%	Excluding breaks, loading and unloading. Fresh produce sometimes operates with two drivers.
Common	Max time in use	h per day	12	15	12	12	0%	Including breaks, loading and unloading.
Common	Power, peak	kW	750.0	550.0	300.0	160.0	0%	Nils-Gunnar Vågstedt, Scania, private communication
Common	Tyres	euro per km	0.113	0.090	0.061	0.031	0%	ASEK 7
Common	Cargo capacity value	euro per ton-km	0.025	0.025	0.035	0.05	0%	Roger Blom, CEO at Ernsts Express, private communication
Common	Cargo capacity value	euro per m3-km	0	0	0	0	0%	280 kg/m3, lost the source
Common	Total weight limit	kg	60000	40000	24000	16000	0%	Definition
Common	Depot stop	h	12	9	12	12	0%	Complement to utilization
Common	Destination stop	h	1	1	0.5	0.2	0%	Guess
Common	Rest stop	h	0.75	0.75	0.75	0.75	0%	Guess
Common	Drive session	h	4.5	4.5	4.5	4.5	0%	EU regulation
Common	Driver cost	euro per h	34	34	30	30	0%	Roger Blom, CEO at Ernsts Express, private communication
ICEV	Chassis weight	kg	23 000	20 000	10 000	5 000	0%	ASEK 7
ICEV	Chassis cost, 2020	euro	259200	235200	148800	91200	0%	ASEK 7, Kapitel 14 Operativa trafikeringskostnader för godstransporter
ICEV	Lifetime	years	10	10	7	7	0%	ASEK 7, Kapitel 14 Operativa trafikeringskostnader för godstransporter
ICEV	Maintenance cost	euro per km	0.100	0.114	0.121	0.102	0%	ASEK 7, Kapitel 14 Operativa trafikeringskostnader för godstransporter
ICEV	Residual value at end of life	ratio	9%	9%	9%	9%	0%	ASEK 7, Kapitel 14 Operativa trafikeringskostnader för godstransporter
ICEV	Fuel consumption	liter per km	0.35	0.27	0.22	0.16	-0.5%	ASEK 7, Kapitel 14 Operativa trafikeringskostnader för godstransporter. See notes below on assumed annual energy consumption improvements.
BEV	Maintenance cost	euro per km	*0.040*	*0.046*	*0.048*	*0.041*	0%	40% av ICEV

BEV	Residual value at end of life	ratio	*9%*	*9%*	*9%*	*9%*	0%	Same as ICEV
BEV	Energy consumption	kWh per km	*1.46*	*1.12*	*0.64*	*0.47*	-1%	Converted from ICEV consumption, diesel energy density and propulsion efficiency difference. See notes below on assumed annual energy consumption improvements. Energy consumption is adjusted at run-time based on vehicle weight relative to ICEV. See Methods.
BEV	Min range buffer	km	50	50	30	20	0%	Guess
BEV	Lifetime 2020	years	*10*	*10*	*7*	*7*		Nils-Gunnar Vågstedt, Scania, and Anders Berger, Volvo Trucks, private communication
BEV	Lifetime 2035	years	15	15	12	12		Nils-Gunnar Vågstedt, Scania, and Anders Berger, Volvo Trucks, private communication
BEV	Energy per day of operation	kWh	*570*	*562*	*223*	*85*		

Supplementary Table 8: HGV60 Parameter Settings

Derived parameter values in MOSTACHI's up to 60-ton heavy goods vehicle (HGV60) category.

Subgroup	variable	unit	2020	2025	2030	2035	2040	2045	2050
Common	Annual distance	km	90 000	90 000	90 000	90 000	90 000	90 000	90 000
Common	Utilization	calendar days per year	230	230	230	230	230	230	230
Common	Max time in transit	h per day	9	9	9	9	9	9	9
Common	Max time in use	h per day	12	12	12	12	12	12	12
Common	Power, peak	kW	750	750	750	750	750	750	750
Common	Tyres	euro per km	0.11	0.11	0.11	0.11	0.11	0.11	0.11
Common	Cargo capacity value	euro per ton-km	0.025	0.025	0.025	0.025	0.025	0.025	0.025
Common	Cargo capacity value	euro per m3-km	0.007	0.007	0.007	0.007	0.007	0.007	0.007
Common	Total weight limit	kg	60000	60000	60000	60000	60000	60000	60000
Common	Depot stop	h	12.0	12.0	12.0	12.0	12.0	12.0	12.0
Common	Destination stop	h	1.0	1.0	1.0	1.0	1.0	1.0	1.0
Common	Rest stop	h	0.75	0.75	0.75	0.75	0.75	0.75	0.75
Common	Drive session	h	4.5	4.5	4.5	4.5	4.5	4.5	4.5
Common	Driver cost	euro per h	34	34	34	34	34	34	34
ICEV	Chassis weight	kg	23 000	23 000	23 000	23 000	23 000	23 000	23 000
ICEV	Chassis cost, 2020	euro	259200	259200	259200	259200	259200	259200	259200
ICEV	Lifetime	years	10.00	10.00	10.00	10.00	10.00	10.00	10.00
ICEV	Maintenance cost	euro per km	0.09984	0.09984	0.09984	0.09984	0.09984	0.09984	0.09984
ICEV	Residual value at end of life	ratio	0.09	0.09	0.09	0.09	0.09	0.09	0.09
ICEV	Fuel consumption	liter per km	0.350	0.341	0.333	0.325	0.317	0.309	0.301
BEV	Maintenance cost	euro per km	0.039936	0.039936	0.039936	0.039936	0.039936	0.039936	0.039936
BEV	Residual value at end of life	ratio	0.09	0.09	0.09	0.09	0.09	0.09	0.09
BEV	Energy consumption	kWh per km	1.5	1.4	1.4	1.3	1.3	1.3	1.2
BEV	Min range buffer	km	50.0	50.0	50.0	50.0	50.0	50.0	50.0
BEV	Lifetime	years	10	12	13	15	15	15	15
ICEV	Chassis cost	euro	259 200	262 737	266 454	270 361	274 467	278 782	283 318
BEV	Chassis weight, excl battery	kg	17 780	17 780	17 780	17 780	17 780	17 780	17 780
BEV	Chassis cost, excl battery	euro	215795	213303	211051	209016	207176	205513	204009
BEV	Min net battery capacity	kWh	217	226	205	177	149	124	102

Supplementary Table 9: HGV40 Parameter Settings

Derived parameter values in MOSTACHI's up to 40-ton heavy goods vehicle (HGV40) category.

subgroup	variable	unit	2020	2025	2030	2035	2040	2045	2050
Common	Annual distance	km	115 000	115 000	115 000	115 000	115 000	115 000	115 000
Common	Utilization	calendar days per year	230	230	230	230	230	230	230
Common	Max time in transit	h per day	11	11	11	11	11	11	11
Common	Max time in use	h per day	15	15	15	15	15	15	15
Common	Power, peak	kW	550.00	550.00	550.00	550.00	550.00	550.00	550.00
Common	Tyres	euro per km	0.090	0.090	0.090	0.090	0.090	0.090	0.090
Common	Cargo capacity value	euro per ton-km	0.025	0.025	0.025	0.025	0.025	0.025	0.025
Common	Cargo capacity value	euro per m3-km	0.007	0.007	0.007	0.007	0.007	0.007	0.007
Common	Total weight limit	kg	40000	40000	40000	40000	40000	40000	40000
Common	Depot stop	h	9.0	9.0	9.0	9.0	9.0	9.0	9.0
Common	Destination stop	h	1.0	1.0	1.0	1.0	1.0	1.0	1.0
Common	Rest stop	h	0.75	0.75	0.75	0.75	0.75	0.75	0.75
Common	Drive session	h	4.5	4.5	4.5	4.5	4.5	4.5	4.5
Common	Driver cost	euro per h	34	34	34	34	34	34	34
ICEV	Chassis weight	kg	20 000	20 000	20 000	20 000	20 000	20 000	20 000
ICEV	Chassis cost, 2020	euro	235200	235200	235200	235200	235200	235200	235200
ICEV	Lifetime	years	10.00	10.00	10.00	10.00	10.00	10.00	10.00
ICEV	Maintenance cost	euro per km	0.11424	0.11424	0.11424	0.11424	0.11424	0.11424	0.11424
ICEV	Residual value at end of life	ratio	0.09	0.09	0.09	0.09	0.09	0.09	0.09
ICEV	Fuel consumption	liter per km	0.270	0.263	0.257	0.250	0.244	0.238	0.232
BEV	Maintenance cost	euro per km	0.045696	0.045696	0.045696	0.045696	0.045696	0.045696	0.045696
BEV	Residual value at end of life	ratio	0.09	0.09	0.09	0.09	0.09	0.09	0.09
BEV	Energy consumption	kWh per km	1.1	1.1	1.1	1.0	1.0	1.0	0.9
BEV	Min range buffer	km	50.0	50.0	50.0	50.0	50.0	50.0	50.0
BEV	Lifetime	years	10	12	13	15	15	15	15
ICEV	Chassis cost	euro	235 200	237 795	240 522	243 388	246 400	249 566	252 893
BEV	Chassis weight, excl battery	kg	16 172	16 172	16 172	16 172	16 172	16 172	16 172
BEV	Chassis cost, excl battery	euro	204866	202893	201110	199499	198042	196725	195534
BEV	Min net battery capacity	kWh	159	166	151	130	109	91	75

Supplementary Table 10: MGV24 Parameter Settings

Derived parameter values in MOSTACHI's up to 24-ton medium goods vehicle (MGV24) category.

subgroup	variable	unit	2020	2025	2030	2035	2040	2045	2050
Common	Annual distance	km	80 000	80 000	80 000	80 000	80 000	80 000	80 000
Common	Utilization	calendar days per year	230	230	230	230	230	230	230
Common	Max time in transit	h per day	9	9	9	9	9	9	9
Common	Max time in use	h per day	12	12	12	12	12	12	12
Common	Power, peak	kW	300.00	300.00	300.00	300.00	300.00	300.00	300.00
Common	Tyres	euro per km	0.061	0.061	0.061	0.061	0.061	0.061	0.061
Common	Cargo capacity value	euro per ton-km	0.035	0.035	0.035	0.035	0.035	0.035	0.035
Common	Cargo capacity value	euro per m3-km	0.010	0.010	0.010	0.010	0.010	0.010	0.010
Common	Total weight limit	kg	24000	24000	24000	24000	24000	24000	24000
Common	Depot stop	h	12.0	12.0	12.0	12.0	12.0	12.0	12.0
Common	Destination stop	h	0.5	0.5	0.5	0.5	0.5	0.5	0.5
Common	Rest stop	h	0.75	0.75	0.75	0.75	0.75	0.75	0.75
Common	Drive session	h	4.5	4.5	4.5	4.5	4.5	4.5	4.5
Common	Driver cost	euro per h	30	30	30	30	30	30	30
ICEV	Chassis weight	kg	10 000	10 000	10 000	10 000	10 000	10 000	10 000
ICEV	Chassis cost, 2020	euro	148800	148800	148800	148800	148800	148800	148800
ICEV	Lifetime	years	7.00	7.00	7.00	7.00	7.00	7.00	7.00
ICEV	Maintenance cost	euro per km	0.12096	0.12096	0.12096	0.12096	0.12096	0.12096	0.12096
ICEV	Residual value at end of life	ratio	0.09	0.09	0.09	0.09	0.09	0.09	0.09
ICEV	Fuel consumption	liter per km	0.220	0.215	0.209	0.204	0.199	0.194	0.189
BEV	Maintenance cost	euro per km	0.048384	0.048384	0.048384	0.048384	0.048384	0.048384	0.048384
BEV	Residual value at end of life	ratio	0.09	0.09	0.09	0.09	0.09	0.09	0.09
BEV	Energy consumption	kWh per km	0.6	0.6	0.6	0.6	0.6	0.6	0.5
BEV	Min range buffer	km	30.0	30.0	30.0	30.0	30.0	30.0	30.0
BEV	Lifetime	years	7	9	10	12	12	12	12
ICEV	Chassis cost	euro	148 800	150 217	151 706	153 271	154 916	156 645	158 462
BEV	Chassis weight, excl battery	kg	7 912	7 912	7 912	7 912	7 912	7 912	7 912
BEV	Chassis cost, excl battery	euro	134805	133481	132284	131202	130224	129340	128541
BEV	Min net battery capacity	kWh	87	90	82	71	60	49	41

Supplementary Table 11: MGV16 Parameter Settings

Derived parameter values in MOSTACHI's up to 16-ton medium goods vehicle (MGV16) category.

subgroup	variable	unit	2020	2025	2030	2035	2040	2045	2050
Common	Annual distance	km	42 000	42 000	42 000	42 000	42 000	42 000	42 000
Common	Utilization	calendar days per year	230	230	230	230	230	230	230
Common	Max time in transit	h per day	9	9	9	9	9	9	9
Common	Max time in use	h per day	12	12	12	12	12	12	12
Common	Power, peak	kW	160.00	160.00	160.00	160.00	160.00	160.00	160.00
Common	Tyres	euro per km	0.031	0.031	0.031	0.031	0.031	0.031	0.031
Common	Cargo capacity value	euro per ton-km	0.05	0.05	0.05	0.05	0.05	0.05	0.05
Common	Cargo capacity value	euro per m3-km	0.014	0.014	0.014	0.014	0.014	0.014	0.014
Common	Total weight limit	kg	16000	16000	16000	16000	16000	16000	16000
Common	Depot stop	h	12.0	12.0	12.0	12.0	12.0	12.0	12.0
Common	Destination stop	h	0.2	0.2	0.2	0.2	0.2	0.2	0.2
Common	Rest stop	h	0.75	0.75	0.75	0.75	0.75	0.75	0.75
Common	Drive session	h	4.5	4.5	4.5	4.5	4.5	4.5	4.5
Common	Driver cost	euro per h	30	30	30	30	30	30	30
ICEV	Chassis weight	kg	5 000	5 000	5 000	5 000	5 000	5 000	5 000
ICEV	Chassis cost, 2020	euro	91200	91200	91200	91200	91200	91200	91200
ICEV	Lifetime	years	7.00	7.00	7.00	7.00	7.00	7.00	7.00
ICEV	Maintenance cost	euro per km	0.10176	0.10176	0.10176	0.10176	0.10176	0.10176	0.10176
ICEV	Residual value at end of life	ratio	0.09	0.09	0.09	0.09	0.09	0.09	0.09
ICEV	Fuel consumption	liter per km	0.160	0.156	0.152	0.148	0.145	0.141	0.138
BEV	Maintenance cost	euro per km	0.040704	0.040704	0.040704	0.040704	0.040704	0.040704	0.040704
BEV	Residual value at end of life	ratio	0.09	0.09	0.09	0.09	0.09	0.09	0.09
BEV	Energy consumption	kWh per km	0.5	0.5	0.4	0.4	0.4	0.4	0.4
BEV	Min range buffer	km	20.0	20.0	20.0	20.0	20.0	20.0	20.0
BEV	Lifetime	years	7	9	10	12	12	12	12
ICEV	Chassis cost	euro	91 200	91 957	92 753	93 590	94 469	95 393	96 365
BEV	Chassis weight, excl battery	kg	3 886	3 886	3 886	3 886	3 886	3 886	3 886
BEV	Chassis cost, excl battery	euro	86355	85394	84525	83740	83030	82389	81809
BEV	Min net battery capacity	kWh	46	48	44	38	32	26	22

Supplementary Table 12: Charging Infrastructure Parameter Settings

Values used for all input parameters in MOSTACHI's "Charging infrastructure" category. Electric road system (ERS) cost parameters are aggregates of independently provided estimates by the hardware providers Siemens (overhead catenary ERS), Elonroad (in-road conductive ERS) and Electreon (in-road inductive ERS) in private communication in 2023. Representatives did not wish to be individually quoted. Cost estimates for all years are for deployment at scale, not pilot installations. Numbers for scaled up infrastructure are inherently speculative as only pilot installations have been made to date. In the neutral scenario with 2,000 km ERS with policy to incentivize participation, installed peak ERS power per bidirectional km is approximately 3 MW/km (bidirectional) on the major Swedish motorways (e.g., E4 between Malmö and Stockholm) with double real traffic. This results in an assumed levelized cost per ERS km of approximately 4 million €/km, plus maintenance and capital interest. Grid fees, electricity and taxes are added when calculating user fees. Supplementary Fig. 2 shows resulting lifetime costs for ERS with a peak charging capacity of 3 MW per km.

subgroup	variable	unit	2020	2025	2030	2035	2040	2045	2050	Annual change	Source
ERS	base cost	euro per km	1 500 000	1 426 485	1 356 573	1 290 088	1 226 860	1 166 732	1 109 551	-1%	See comment
ERS	power cost	euro per kW-km	700	666	633	602	573	544	518	-1%	See comment
ERS	maintenance cost	ratio per year	0.02	0.02	0.02	0.02	0.02	0.02	0.02	0%	See comment
ERS	write off period	years	25	25	25	25	25	25	25	0%	See comment
ERS	profit margin	ratio	10%	10%	10%	10%	10%	10%	10%	0%	Same as station
ERS	pick up cost base light	euro	500	452	409	369	334	302	273	-2%	See comment
ERS	pick up cost base heavy	euro	2 000	1 808	1 634	1 477	1 335	1 207	1 091	-2%	See comment
ERS	pick-up cost	euro per kW	50	45	41	37	33	30	27	-2%	See comment
ERS	pick-up lifespan	years	10	10	10	10	10	10	10	0%	See comment
ERS	pick up weight light	kg	50	48	45	43	41	39	37	-1%	See comment
ERS	pick up weight heavy	kg	300	285	271	258	245	233	222	-1%	See comment
ERS	standardization risk	ratio	50%	16%	5%	2%	1%	0%	0%	-20%	Risk of having picked a different standard than neighboring countries
ERS	cost of standard change	ratio	30%	30%	30%	30%	30%	30%	30%	0%	Cost of retrofitting a new standard
ERS	utilization	ratio	27%	27%	27%	27%	27%	27%	27%	0%	
ERS	electricity price	ratio	73%	73%	73%	73%	73%	73%	73%	0%	
ERS	grid connection cable	euro	10 000	10 000	10 000	10 000	10 000	10 000	10 000	0%	20 euro/m, 500 m (All digging costs from: https://www.diva-portal.org/smash/get/diva2:207284/FULLTEXT01.pdf)
ERS	grid connection interval	km	10	10	10	10	10	10	10	0%	See comment
ERS	reference	aadt	2000	2000	2000	2000	2000	2000	2000	0%	Bidirectional AADT Heavy in Sweden is ~4k on the largest roads. Assume half use ERS.
Rest Stop	hardware cost	euro per kW	300	285	271	258	245	233	222	-1%	300 from Andreas Kammel (personal communication). 500 (probably includes grid) from a mix of Trafikverket, Karlström 2020 ("Kunskapssammanställning stationär laddning till tunga lastbilar"), Scania offer, Future Energy (FE). FE clearly states that hardware cost per kW increases with higher power.
Rest Stop	hardware maintenance	ratio per year	10%	10%	10%	9%	9%	9%	9%	-1%	Guess based on discussions with ChargeNode
Rest Stop	grid connection cable	euro	15 000	15 000	15 000	15 000	15 000	15 000	15 000	0%	20 euro/m, 750 m
Rest Stop	write off period	years	15	15	15	15	15	15	15	0%	TRV says 25. Kristoffer Ahlén, Price and Product Manager at Preem, says 10.
Rest Stop	profit margin	ratio	10%	10%	10%	10%	10%	10%	10%	0%	Guess
Rest Stop	utilization	ratio	20%	20%	20%	20%	20%	20%	20%	0%	
Rest Stop	electricity price	ratio	73%	73%	73%	73%	73%	73%	73%	0%	
Rest Stop	reference	vehicles per day	100	100	100	100	100	100	100	0%	Seed value. Bidirectional AADT Heavy in Sweden is ~4k on the largest roads. If vehicles must stop every 5th opportunity and half use stations.
Depot	hardware cost	euro per kW	300	285	271	258	245	233	222	-1%	0

Depot	hardware maintenance	ratio per year	10%	10%	10%	9%	9%	9%	9%	-1%	Guess based on discussions with ChargeNode
Depot	grid connection cable	euro	5 000	5 000	5 000	5 000	5 000	5 000	5 000	0%	50 euro/m, 100 m
Depot	write off period	years	7	7	7	7	7	7	7	0%	Guess
Depot	profit margin	ratio	10%	10%	10%	10%	10%	10%	10%	0%	Guess, haulier rents infra
Depot	utilization	ratio	34%	34%	34%	34%	34%	34%	34%	0%	
Depot	electricity price	ratio	38%	38%	38%	38%	38%	38%	38%	0%	
Depot	reference	vehicles per day	20	20	20	20	20	20	20	0%	Seed value. Guess.
Destination	hardware cost	euro per kW	300	285	271	258	245	233	222	-1%	0
Destination	hardware maintenance	ratio per year	10%	10%	10%	9%	9%	9%	9%	-1%	As rest stop
Destination	grid connection cable	euro	5 000	5 000	5 000	5 000	5 000	5 000	5 000	0%	As depot
Destination	write off period	years	10	10	10	10	10	10	10	0%	Guess
Destination	profit margin	ratio	10%	10%	10%	10%	10%	10%	10%	0%	As depot
Destination	utilization	ratio	13%	13%	13%	13%	13%	13%	13%	0%	
Destination	electricity price	ratio	82%	82%	82%	82%	82%	82%	82%	0%	
Destination	reference	vehicles per day	50	50	50	50	50	50	50	0%	Seed value. Guess.
Grid	initial acccess base	euro	5 000	5 000	5 000	5 000	5 000	5 000	5 000	0%	Figure from Ellevio (Swedish DSO) used in past project
Grid	initial acccess	euro per kW	300	300	300	300	300	300	300	0%	As above. Adjusted from 100 to 300 based on feedback from Andreas Kammel.
Grid	fee at 10 percent utilization	euro per kWh	0.06	0.06	0.06	0.06	0.06	0.06	0.06	0%	As above

Supplementary Table 13: Infrastructure Utilization Curve Parameter Settings

Calculation of charging infrastructure utilization fees (€/kWh) requires knowledge of infrastructure utilization rates. However, the input transport route data lacks information about when a route is traversed. Therefore, utilization rates are estimated globally, per type of charging infrastructure. Energetic utilization rate is estimated per site or ERS km (not outlet) and is defined here as [annual energy delivered] / ([peak installed power] * [hours per year]). MOSTACHI does not capture well the effects that increasing density of battery-electric truck (BET) traffic and declining costs of stationary energy storage will have on energetic charging infrastructure utilization. Therefore, the annual utilization figures in the table above were altered for each simulation year to be [35%, 35%, 35%, 45%, 60%, 80%, 100%] of the final value for years 2020—2050. This raises the levelized cost of the charging infrastructure itself in the earlier years of the simulation.

Hour of day	Depot	Destination	Rest Stop	ERS
0	100%	0%	15%	15%
1	100%	0%	15%	15%
2	100%	0%	15%	15%
3	100%	0%	15%	15%
4	100%	0%	20%	20%
5	85%	0%	30%	30%
6	70%	0%	50%	50%
7	30%	20%	60%	60%
8	10%	50%	75%	75%
9	5%	100%	90%	90%
10	5%	100%	100%	100%
11	5%	100%	100%	100%
12	80%	70%	100%	100%
13	80%	70%	100%	100%
14	5%	100%	100%	100%
15	5%	100%	90%	90%
16	5%	100%	80%	80%
17	30%	50%	70%	70%
18	70%	20%	60%	60%
19	80%	0%	40%	40%
20	90%	0%	30%	30%
21	100%	0%	20%	20%
22	100%	0%	15%	15%
23	100%	0%	15%	15%
**Average**	*61%*	*37%*	*54%*	*54%*

Day of week	Utilization
Mon	100%
Tue	100%
Wed	100%
Thu	100%
Fri	100%
Sat	40%
Sun	40%
**Average**	*83%*

**Month**	**Utilization**
Jan	80%
Feb	100%
Mar	100%
Apr	100%
Maj	100%
Jun	80%
Jul	40%
Aug	80%
Sep	100%
Okt	100%
Nov	100%
Dec	80%
**Average**	*88%*

variable	unit	Depot	Destination	Rest Stop	ERS
Overcapacity (demand variability)	percent	*30%*	*100%*	*100%*	*50%*
Annual utilization	percent	*34%*	*13%*	*20%*	*27%*
Electricity price	ratio min to max	*0.376*	*0.825*	*0.726*	*0.726*
Grid fee	euro per kWh	*0.018*	*0.045*	*0.030*	*0.023*
Electricity price €/kWh	euro per kWh	*0.092*	*0.116*	*0.110*	*0.110*

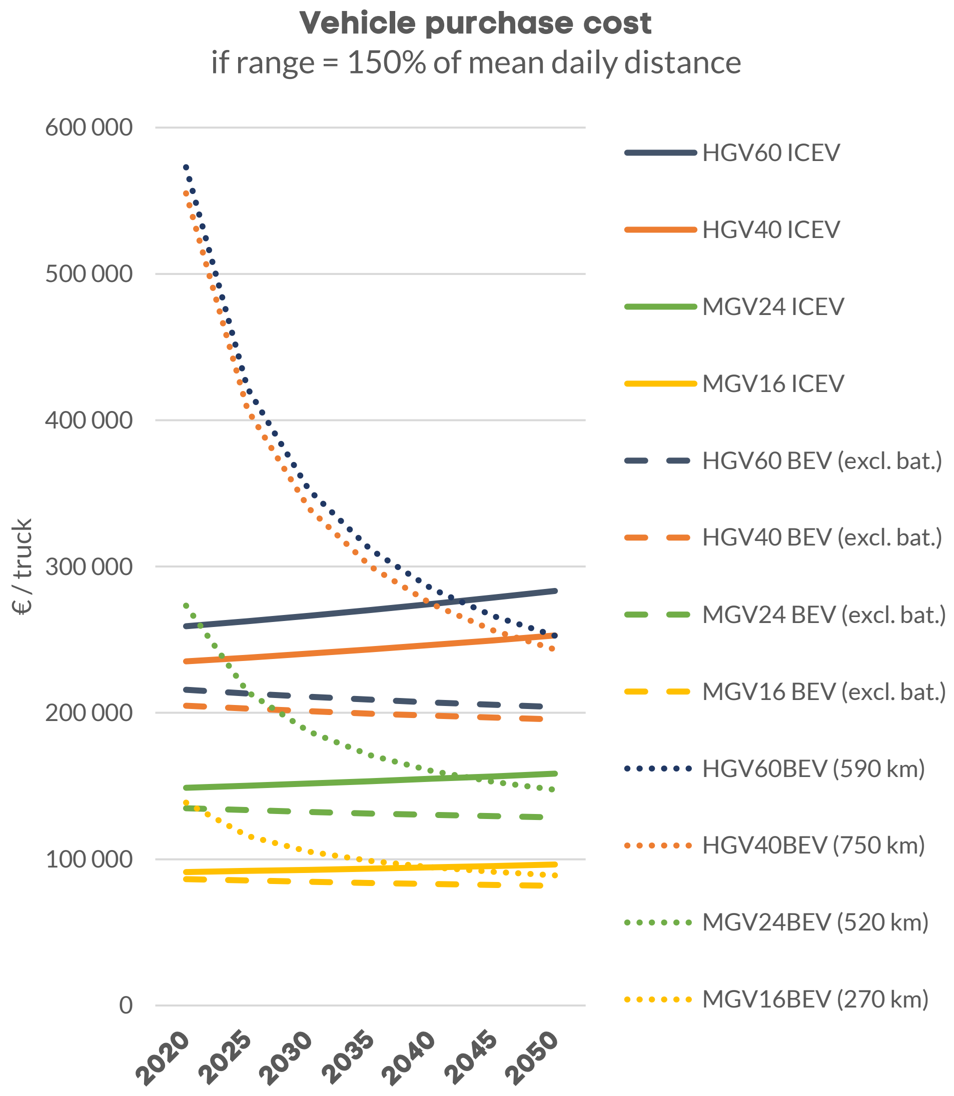

Supplementary Figure 1: BET Purchase Cost

The figure below shows effective battery-electric truck (BET) costs using these parameters if batteries are sized to allow a full day of operation on a single charge, most days. Well-optimized charging infrastructure enables the same operation to be performed with a smaller battery, which reduces BET cost.

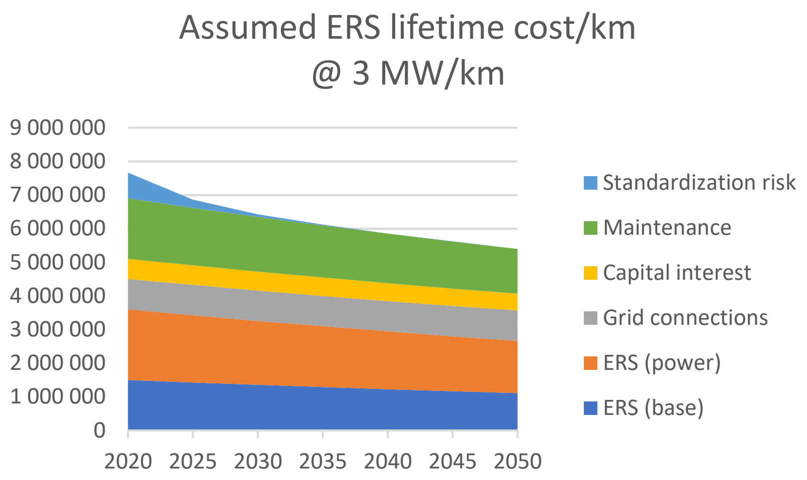

Supplementary Figure 2: ERS Cost

The resulting non-discounted lifetime cost per installed distance of electric road systems (ERS) when combining all default model parameters.

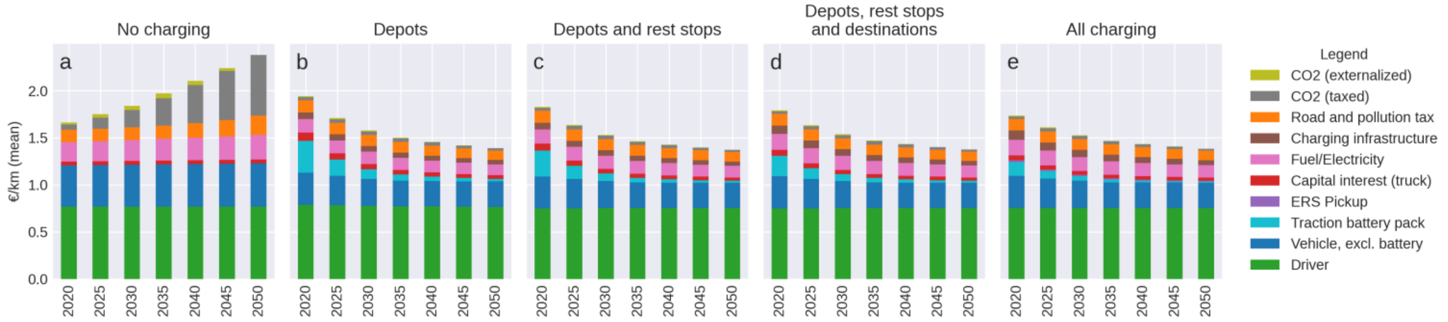

Supplementary Figure 3: Itemized system costs

The figure shows distance-levelized system costs (€/km) resulting from optimization using neutral global parameters and unlimited charging infrastructure of different types. Diesel costs were set to 10x default values in scenarios with charging infrastructure (panels b-e) to force all routes to use electric vehicles. Other model parameters match the neutral scenario in the study. The largest single cost is always driver salaries, followed by vehicles. Total system cost depends almost exclusively on the share of transport that is electrified. Without charging infrastructure (a), transport along all routes is performed with combustion engine vehicles. The set of available infrastructure has very limited impact on total system cost when infrastructure is unrestricted, in particular from 2030 and onwards. More charging infrastructure makes it possible to complete transport operations with traction battery packs with lower capacity, which reduces costs. Panels b-e show how offering additional charging infrastructure causes a transfer of costs from batteries to infrastructure, resulting in a small net reduction in total costs.

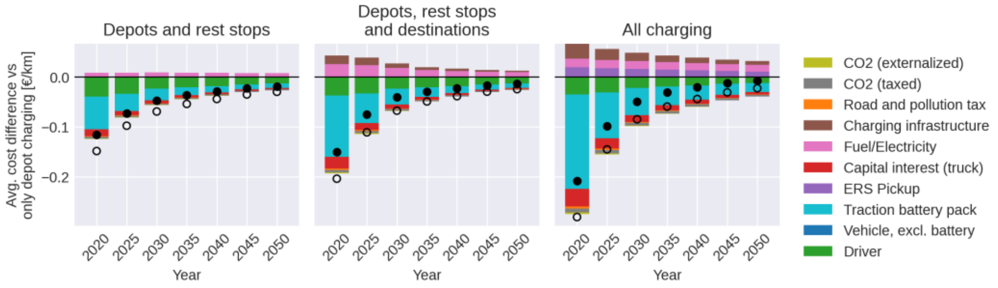

Supplementary Figure 4: Infrastructure impact on levelized BEV costs

The figure uses the same data as in Supplementary figure 3, but shows only the change in distance-levelized system costs (€/km) with increasing availability of charging types, versus if charging is only available at depots. Positive bars indicate an increase in costs and negative bars indicate a decrease in costs. Filled black circles indicate the sum of differences. Note that changes in vehicle weight and charging patterns can affect the number of vehicle trips required to transport a fixed quantity of goods along a route, which affects total operating costs but not the levelized cost per vehicle km. Hollow circles indicate sum of cost differences after compensating for changes in total driven distance. Future work is recommended to explore dynamics that are not captured in MOSTACHI, as these may have greater impact on long-term charging preferences than the (very minor) differences in modelled costs. Conditions on individual routes or under different scenarios can differ significantly from the system averages in this figure.

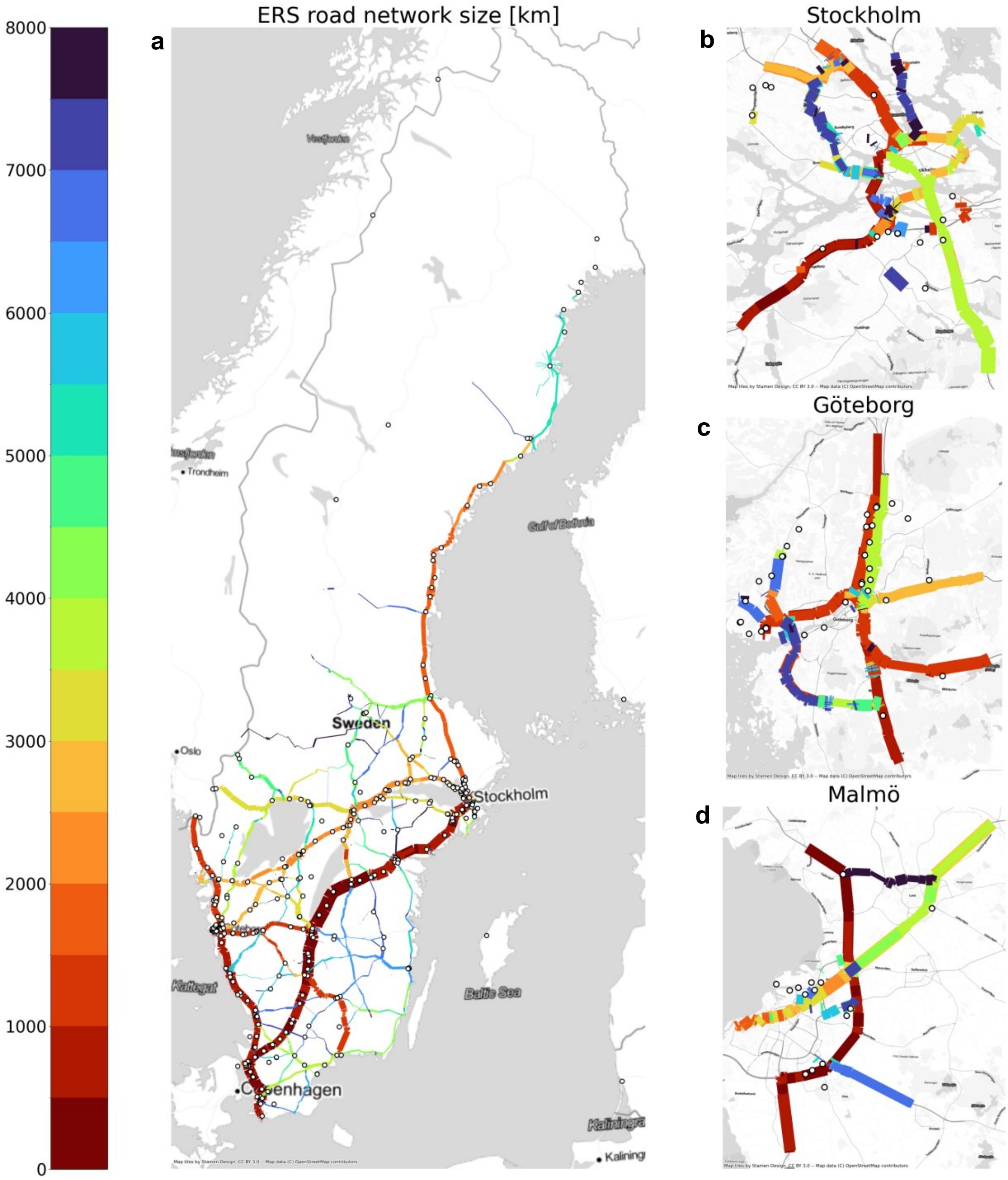

Supplementary Figure 5: Rest stop and ERS placement

Colors (red to blue) indicate the precalculated order of electric road system (ERS) buildout identified using Supplementary Method 1. Panel a shows the first 8,000 km of the full road network, with panels b-d showing close-up views of road segments contained within bounding boxes around the three most populous cities. E.g., in a scenario that offers 2,000 km of ERS, all segments (gaps excluded) from red to dark orange are offered and the optimization selects which of these will be built. Small black circles indicate the locations of rest areas, where public fast charging infrastructure can be placed. The order of buildout at rest stops is random, but consistent between scenarios. Base map data from OpenStreetMap, licensed under CC BY-SA 2.0, and map tiles from Stamen Design, licensed under CC BY 3.0.

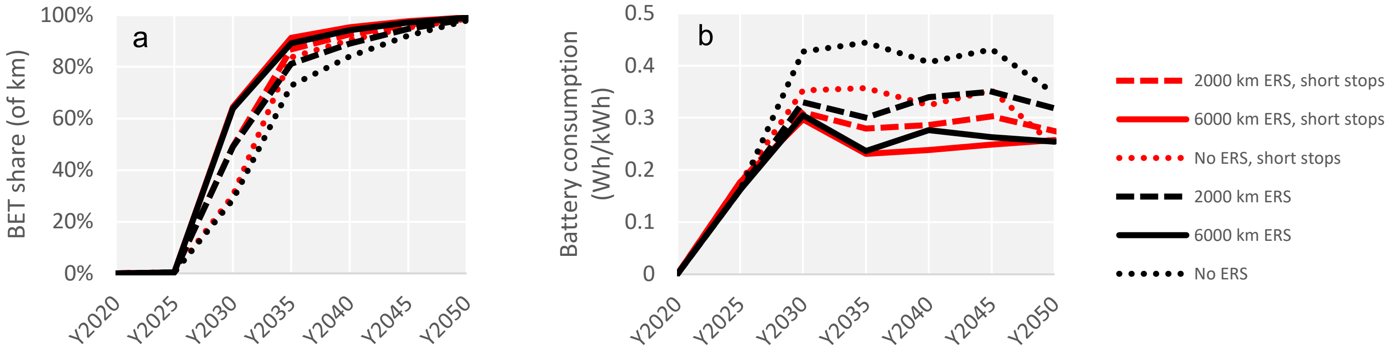

Supplementary Figure 6: Effects of taking shorter and more frequent rest stops

A reviewer directed our attention to a prior study that concluded the battery capacity in trucks can be reduced if drivers split their mandated 45-minute break into two shorter breaks, as permitted within the European Union. See reference in Discussion. To verify the robustness of our own findings, we have run additional experiments in MOSTACHI, using the Neutral condition with 0, 2,000 and 6,000 km electric road system (ERS), with policy support for ERS, and with/without shortened stops. Scenarios with shortened stops were simulated with up to 4.5x0.5 h drive time between rest stops, and 45x0.5 minutes rest stop duration. A small overhead cost (time) for all rest stops is included in the simulation, representing the combined effects of detours, queueing for charging, plugging in, etc. The figure shows that short frequent stops have a net positive effect on battery-electric truck (BET) uptake (panel a), which stacks with the positive effect from ERS. Our simulations also confirm the finding from prior work that more frequent but shorter stops enable battery capacity reductions, at approximately the ratios identified in the earlier study. This has been quantified at system level (panel b) as gross battery storge capacity spent per unit of cycled energy, in Wh/kWh (the full battery capacity is considered spent at the end of the vehicle lifetime). As with BET uptake, there is a compounding effect with ERS. We have not identified that any of the main findings of our research change when shorter stops are simulated.

Supplementary Method 1: Calculation of the build-out order of segments in the ERS network

The following algorithm, in pseudo-code, was used to calculate the electric road system (ERS) build-out order shown in supplementary figure 4.

```
Create an index I_A:(S_a,S_b)→W_ab, b>a of association strength W_ab between pairs of road segments (S_a,S_b)∈S
Create an index I_T:R_x→T_x of total number of annual trips T_x for each route R_x∈R
Discard all R_x with trip count T_x < 150 or segment sequence length |S_R| < 100
Build an index I_R:C_a→(C_b,S_x[])[] of all routes R_0 that go between two grid cells (C_a,C_b)∈C
For each grid cell C_i, in parallel:
... For each route R_j departing from C_i:
... ....Make a set S of all the segments traversed by R_i
... ....If a route R_r pointing in the opposite direction to R_j exists:
... .... ...Get R_r from I and add the segments in R_r to S, with repetition
... ....Take a random sample S* of 100 road segments from S
... ....Remove repeating items from S*
... ....For each pairwise combination (S_a,S_b) of segments in S*:
... .... ...Increment the association strength W_x in I_A for (S_a,S_b) by T_x from I_T
... ....After every 10k pairs of (C_a,R_a), pause the parallel loop and:
... .... ...Discard all (S_a,S_b)∈I_A where W_ab < max(W)/1000
Discard all (S_a,S_b)∈I_A where W_ab < 2000
For each segment S_a in I_A:
... Discard all but the top 100 associations (S_a,S_b) from I_A in descending order by W_ab
Build a graph G representing the node-edge relationship of I_A:
... Edges E_ab∈E represent a road segment pair (S_a,S_b) with weight W_ab
... Set E^a∈E contains all edges connected to node S_a
... Set E^m∈E^a is the subset of edges that connect S_a to another already marked segment S_b
... Nodes S_a∈S represent road segments, with properties V_a, V_a*: and marked∈[true, false]
... ....V_a = ∑W_ab over E^a (kept constant)
... ....V_a* = log(V_a) * ∑W_ab over E^m (updates when a neighbor gets marked)
... ....V_x* is initialized to a random number r∈[0,1]
Start with a seed of marked road segments
Create a list L of all nodes, sorted in ascending order of V_x*
Create a list O containing the segment build our order
While |L|>0:
... Mark the last item S_x in L and remove it from L
... Append S_x to O
... Resort L
```
