# Supplementary material for: Correcting market failure for no-regret electric road investments under uncertainty
Source: Nat Commun. 2025 Aug 11;16:7398. doi: 10.1038/s41467-025-62679-w (PMC12340124; doi:10.1038/s41467-025-62679-w)
Supplement: Supplementary file 2 — Reporting Summary [file 41467_2025_62679_MOESM2_ESM.pdf]

nature portfolio

Corresponding author(s): Jakob Rogstadius

Last updated by author(s): Jun 27, 2025

Reporting Summary

Nature Portfolio wishes to improve the reproducibility of the work that we publish. This form provides structure for consistency and transparency in reporting. For further information on Nature Portfolio policies, see our Editorial Policies and the Editorial Policy Checklist.

Statistics

For all statistical analyses, confirm that the following items are present in the figure legend, table legend, main text, or Methods section.

n/a	Confirmed	
☐	☒	The exact sample size (n) for each experimental group/condition, given as a discrete number and unit of measurement
☐	☒	A statement on whether measurements were taken from distinct samples or whether the same sample was measured repeatedly
☒	☐	The statistical test(s) used AND whether they are one- or two-sided<br>*Only common tests should be described solely by name; describe more complex techniques in the Methods section.*
☐	☒	A description of all covariates tested
☐	☒	A description of any assumptions or corrections, such as tests of normality and adjustment for multiple comparisons
☐	☒	A full description of the statistical parameters including central tendency (e.g. means) or other basic estimates (e.g. regression coefficient) AND variation (e.g. standard deviation) or associated estimates of uncertainty (e.g. confidence intervals)
☒	☐	For null hypothesis testing, the test statistic (e.g. F, t, r) with confidence intervals, effect sizes, degrees of freedom and P value noted<br>*Give P values as exact values whenever suitable.*
☒	☐	For Bayesian analysis, information on the choice of priors and Markov chain Monte Carlo settings
☒	☐	For hierarchical and complex designs, identification of the appropriate level for tests and full reporting of outcomes
☒	☐	Estimates of effect sizes (e.g. Cohen's d, Pearson's r), indicating how they were calculated

Our web collection on statistics for biologists contains articles on many of the points above.

Software and code

Policy information about availability of computer code

Data collection	The study is based on simulations with a custom software tool. The software version used for this study is available at https://github.com/JakobRogstadius/MOSTACHI/releases/tag/v1.0.4 and separately archived at https://doi.org/10.5281/zenodo.15755307. All source code, input and output data are included in the repository. The latest version of the simulation tool is available at https://github.com/JakobRogstadius/MOSTACHI/.
Data analysis	Data analysis was performed on simulation output (log files). Restructured data used to generate all plots in the main manuscript are provided as Source Data, but not the python scripts used to render the figures in the paper.

For manuscripts utilizing custom algorithms or software that are central to the research but not yet described in published literature, software must be made available to editors and reviewers. We strongly encourage code deposition in a community repository (e.g. GitHub). See the Nature Portfolio guidelines for submitting code & software for further information.

Data

Policy information about availability of data

All manuscripts must include a data availability statement. This statement should provide the following information, where applicable:

- Accession codes, unique identifiers, or web links for publicly available datasets
- A description of any restrictions on data availability
- For clinical datasets or third party data, please ensure that the statement adheres to our policy

The input datasets and raw simulation output logs generated in this study have been deposited in the Zenodo database under accession code 1575530739 (https://doi.org/10.5281/zenodo.15755307). The reorganized data used to draw the figures in this study are provided in the Source Data file.

Research involving human participants, their data, or biological material

Policy information about studies with human participants or human data. See also policy information about sex, gender (identity/presentation), and sexual orientation and race, ethnicity and racism.

Reporting on sex and gender	n/a
Reporting on race, ethnicity, or other socially relevant groupings	n/a
Population characteristics	n/a
Recruitment	n/a
Ethics oversight	n/a

Note that full information on the approval of the study protocol must also be provided in the manuscript.

Field-specific reporting

Please select the one below that is the best fit for your research. If you are not sure, read the appropriate sections before making your selection.

☐ Life sciences ☐ Behavioural & social sciences ☒ Ecological, evolutionary & environmental sciences

For a reference copy of the document with all sections, see nature.com/documents/nr-reporting-summary-flat.pdf

Ecological, evolutionary & environmental sciences study design

All studies must disclose on these points even when the disclosure is negative.

Study description	Scenarios were simulated and model outputs compared with different amounts of different types of charging infrastructure for heavy-duty trucks.
Research sample	See "Experimental design". Three datasets are used that were generated with the custom simulation software. 1) simulation results from five parameter conditions with five scenarios in each; 2) a factorial design with 513 scenarios; 3) 1500*2 scenarios using Monte Carlo sampling of model parameters, for sensitivity analysis.
Sampling strategy	The spatial scope of the simulations is the Swedish road network. Of all road traffic, only heavy truck flows were analyzed, and these were taken from a validated national model, not directly measured. The selection of what scenarios to simulate, and the motivation behind these choices, is described in the paper.
Data collection	Simulation output. Simulations use a weighted origin-destination matrix representing traffic intensity between location pars as input, which is itself generated by a validated national model (Samgods) that uses a goods flow survey as its input.
Timing and spatial scale	Scenarios are simulated in five year intervals, at a spatial resolution of road segments of typical length 0.1-10 km.
Data exclusions	The simulations include only traffic with medium- and heavy-duty trucks on transport routes intersecting Sweden. Transport patterns for light trucks were provided, but deemed to have too low quality to include in the study.
Reproducibility	The simulations are nearly deterministic and all data can be fully reproduced by others.
Randomization	n/a
Blinding	n/a

Did the study involve field work? ☐ Yes ☒ No

Reporting for specific materials, systems and methods

We require information from authors about some types of materials, experimental systems and methods used in many studies. Here, indicate whether each material, system or method listed is relevant to your study. If you are not sure if a list item applies to your research, read the appropriate section before selecting a response.

Materials & experimental systems

n/a	Involved in the study
☒	☐ Antibodies
☒	☐ Eukaryotic cell lines
☒	☐ Palaeontology and archaeology
☒	☐ Animals and other organisms
☒	☐ Clinical data
☒	☐ Dual use research of concern
☒	☐ Plants

Methods

n/a	Involved in the study
☒	☐ ChIP-seq
☒	☐ Flow cytometry
☒	☐ MRI-based neuroimaging

Plants

Seed stocks	n/a
Novel plant genotypes	n/a
Authentication	n/a
